# Epidemiological characteristics and spatiotemporal analysis of scarlet fever in Guangzhou, China: the surveillance of 20 years

**DOI:** 10.1186/s12879-026-13770-0

**Published:** 2026-06-26

**Authors:** Hong-rou Lin, Jiangrong Wang, Jiamin Shan, Qing He, Shuwen Dong, Qiang Deng, Meiqi Zhang, Siyu Chen, Lei Luo, Yilan Li, Yanhui Liu

**Affiliations:** 1 Guangzhou Liwan District Center for Disease Control and Prevention (Guangzhou Liwan District Health Supervision Institute), Guangzhou, 510176 China; 2 Guangzhou Center for Disease Control and Prevention (Guangzhou Health Supervision Institute), Guangzhou, 510440 China; 3 Institute of Public Health, Guangzhou Medical University & Guangzhou Center for Disease Control and Prevention, Guangzhou, 510440 China; 4 Department of Infectious Disease Prevention and Control, Guangzhou Center for Disease Control and Prevention (Guangzhou Health Supervision Institute), Guangzhou, 510440 China; 5 Department of School Health, Guangzhou Center for Disease Control and Prevention (Guangzhou Health Supervision Institute), Guangzhou, 510440 China

**Keywords:** Scarlet fever, Epidemiological features, Spatial autocorrelation, Spatial clustering

## Abstract

**Background:**

This study investigated the epidemiological and spatiotemporal characteristics of scarlet fever in Guangzhou, China, and provided a scientific basis for the formulation and optimization of prevention and control strategies.

**Methods:**

The epidemiological profile of scarlet fever was systematically reviewed using descriptive analysis. We used the Moran’s I, local indicators of spatial association, and the retrospective spatiotemporal scanning statistical analysis to study the spatiotemporal aggregation phenomenon of scarlet fever in Guangzhou.

**Results:**

From 2005 to 2024, 9,601 scarlet fever cases were reported within Guangzhou, with an annual average morbidity of 3.16 per 100,000. Joinpoint regression analysis indicated a statistically significant overall upward trend in scarlet fever incidence (AAPC = 17.26%, *p* < 0.001), characterized by four phases: low-incidence phase, upsurge phase, COVID-19-affected phase and post COVID-19 rebound phase. Seasonal distribution exhibited a dual-modal pattern, and the crest of transmission occurred from April to June and November to the following January. Demographically, cases mainly occurred among males and kindergarten children aged 3–6 years. Notably, during the post COVID-19 rebound phase, the proportion of cases among students, particularly those aged 7–10 years, increased substantially, becoming a major affected group nearly comparable to kindergarten children. Spatially, higher incidence was observed within central urban districts and expanding peri-urban growth centers. Spatiotemporal scanning identified ten statistically significant clusters, characterized by an expansion toward expanding peri-urban growth centers, such as Licheng in Zengcheng, alongside a resurgence in traditional central urban areas like Meihuacun in Yuexiu, Baihedong in Liwan and their surrounding regions.

**Conclusions:**

This study reveals a significant spatial-temporal clustering of scarlet fever in Guangzhou from 2005 to 2024, characterized by an overall upward trend and a notable recent resurgence. The high-risk regions were predominantly localized within the central urban regions and expanding peri-urban growth centers, such as Helong, Licheng, Meihuacun, and their surrounding regions. The outbreak mostly affects kindergarten children aged 3–6 years and students aged 7–10 years, with winter and spring as peaks. Control strategies should target high-risk seasons, populations and regions to reduce scarlet fever cases.

**Supplementary Information:**

The online version contains supplementary material available at https://doi.org/10.1186/s12879-026-13770-0.

## Introduction

Scarlet fever is an acute respiratory infectious disease stemming from infection with Group A Streptococcus (GAS, also known as Group A Beta-hemolytic streptococcus) [1, 2], characterized by fever, pharyngitis, bright red small rash scattered all over the body, and obvious desquamation during the recovery period [3]. Severe or untreated cases may progress to pyogenic complications, like otitis media and peritonsillar abscess, or non-suppurative autoimmune disorders, like acute rheumatic fever and streptococcal glomerulonephritis, which may be fatal in rare cases, posing a significant threat to public health, particularly among pediatric populations [4, 5]. Primarily transmitted via respiratory droplets, the disease frequently outbreaks in crowded places such as kindergartens and schools, particularly during peak seasons in winter and spring [6]. In the absence of an approved vaccine [7, 8], the implementation of the “early detection, diagnosis, and treatment” strategy, as well as timely isolation and antibacterial treatment, remain the key control strategy at present [9].

Globally, scarlet fever has exhibited complex epidemiological characteristics over the past century. In the second half of the 20th century, the incidence rate was at a low level in most developed countries due to the widespread use of antibiotics [10, 11]. However, the disease has undergone a resurgence since 2010 across North America, Europe (such as the United Kingdom and Germany), and Asia (such as South Korea and Singapore) [3, 12–16]. Moreover, GAS-related infections impose a notable burden on global health. Annually, GAS pharyngitis affected approximately 616 million individuals globally, and more than 517,000 deaths resulted from GAS infections [17]. The incidence rate of pediatric GAS pharyngitis is approximately 22.10 episodes per 100 child-years [18], and further emphasized that scarlet fever and other diseases related to GAS are major public health problems worldwide.

In China, the incidence of scarlet fever has exhibited a general fluctuating upward trend since 2005 [11], historically following a distinct quadrennial periodicity, with epidemic peaks recorded in 2007, 2011, 2015, and 2019 [19]. The cyclical pattern was notably interrupted during the COVID-19 pandemic, as the implementation of non-pharmaceutical interventions (NPIs), including social distancing and school closures, sustained scarlet fever morbidity at a comparatively low level [20]. However, a substantial resurgence in 2024 signals a significant rebound after the COVID-19 pandemic, and the overall epidemiological trend is on the rise [19, 21]. As a Class B notifiable disease [19], scarlet fever ranked ninth among all Class A and B notifiable infectious diseases in 2022, which highlighted its increasing importance in the field of public health in China [22].

National surveillance data indicated that the incidence of scarlet fever is unevenly distributed in China, mainly concentrated in major megacities and port areas [19]. As a primary economic center and strategic port city in southern China, Guangzhou is characterized by high population density and intense population mobility. These factors may contribute to prominent epidemiological characteristics and a high disease burden, positioning Guangzhou as a critical sentinel region for investigating the transmission dynamics of scarlet fever in China. Although previous studies have documented the epidemiological patterns of scarlet fever in Guangzhou [23, 24], they are limited by short time series, lacking the full 20-year 2005–2024 cycle, and relying primarily on simple descriptive analyses of epidemiological features. Furthermore, most relevant studies in China have only been conducted at the district or county level, with few fine-scale analyses covering all subdistricts and towns across the entire city. Identifying the shifting geographic burden is essential for optimizing public health resource allocation. To date, no study has focused on the spatial and temporal distribution patterns of scarlet fever in Guangzhou, particularly at the fine-grained subdistrict (town) level. Therefore, we utilized the cumulative data of scarlet fever cases and populations in Guangzhou over the past 20 years to investigate the above topic through descriptive analysis, spatial autocorrelation analysis and space-time scanning statistical analysis. The result of the research could offer a stronger scientific basis for the formulation of Guangzhou’s prevention and control plans, strategies and measures for the scarlet fever.

## Methods

### Study area

Guangzhou is a core megacity in the southern China and the capital of Guangdong province. Covering a total landmass of 7,434.40 square kilometers, the region is bounded by the coordinates 112°57′-114°3′E and 22°26′-23°56′N. Guangzhou comprises 11 administrative districts, which are further subdivided into 143 subdistricts and 34 towns (see Supplementary Fig. 1). At the end of 2024, the permanent resident population amounted to 18.978 million people. The climate is a subtropical monsoon type with marked marine impacts, featuring long, hot, rainy summers and short, mild, dry winters.

### Data sources

#### Epidemiological data sources

This study employed a population-based retrospective ecological design with the characteristics of descriptive and quantitative research methods. Between January 1, 2005, and December 31, 2024, the data of scarlet fever cases in Guangzhou were sourced via the National Notifiable Infectious Disease Surveillance System (NNIDSS), which included age, gender, address, date of symptom onset, occupation, and case classification. The centers for disease control and prevention conduct regular supervision and inspection of the reporting situation of infectious diseases every year to ensure that every diagnosed notifiable disease, including scarlet fever, can be reported. This study included clinical diagnosis cases and laboratory confirmed cases, and excluded suspected cases to ensure the accuracy of diagnosis. Finally, 9,601 valid cases were retained for analysis, and no cases have been eliminated due to lack of demographic or geographic information. To ensure patient confidentiality, all personal identifiers were de-identified prior to data analysis. Demographic variables included gender and age, with the latter categorized into five groups to reflect different social and educational settings: prenursery (0–2 years), kindergarten (3–6 years), primary school (7–10 years), middle school (11–14 years), and others (≥15 years). Additionally, occupational categories were grouped into non-enrolled children, kindergarten children, students, and others. Non-enrolled children (aged 0–2 years) are those cared for by family members or nannies at home. Kindergarten children (aged 3–6 years) refer to those enrolled in preschool or childcare institutions. Students (aged 7 years and older) include individuals attending primary school through college. Others primarily encompass the population outside the education system, including individuals in the workforce (e.g., commercial services, manual labor, and healthcare professionals), those engaged in housework, the unemployed, and retirees.

#### Population data sources

Annual population totals and district-level population counts for Guangzhou from 2005 to 2024 were retrieved via the municipal bureau of statistics’ online yearbooks (https://tjj.gz.gov.cn/stats_newtjyw/zyxz/tjnjdzzz/). The demographic data at the subdistrict (town) level was obtained from the WorldPop (https://www.worldpop.org/) at a spatial resolution of 100 meters [25]. The raster population data was summarized to the subdistrict (town) level using zonal statistics to estimate population size for each subdistrict or town. The original estimates of the subdistrict (town) level were further calibrated using official district-level demographic statistics to ensure consistency with the authoritative district-level data.

#### Administrative boundary data sources

Administrative boundary polygons of Guangzhou at district-level and subdistrict (town)-level were retrieved from OpenStreetMap using the Overpass Turbo tool (https://overpass-turbo.eu/), and all boundaries were unified to the WGS84 (EPSG: 4326) spatial reference system to match population and incidence datasets. The extracted boundaries were verified according to the official administrative map of the Guangzhou Municipal Planning and Natural Resources Bureau to ensure that they were consistent with the official administrative division of the city for subsequent spatial analysis.

### Statistical analysis

The incidence rate refers to the number of new cases per 100,000 people, which was calculated by dividing the number of cases per year or month by the corresponding population. Joinpoint regression analysis was conducted using Joinpoint software (version 5.4.0) with a log-linear model to assess temporal trends and detect non-linear shifts (joinpoints) within long-term incidence data. We employed the annual percent change (APC) and average annual percent change (AAPC) to characterize the temporal variation in scarlet fever incidence. The research period was divided into four stages (2005 – 2016, 2017 – 2019, 2020–2022, and 2023 – 2024), which is based on the turning point identification of joint points, annual incidence fluctuations and major public health milestones (especially COVID-19 interventions). We also analyzed the monthly incidence trend to assess the seasonal changes of scarlet fever. Our method included year-to-year comparison to capture the differences in the peak incidence between years, followed by cumulative monthly analysis to determine the overall seasonal pattern for the entire study period. The data processing, analysis, and visualization were performed utilizing Microsoft Excel 2024 and R software version 4.4.1. We set the significance threshold (α) at 0.05 for all statistical tests.

Spatial analysis was conducted at the subdistrict or town level, while temporal analysis was based on years. We conducted spatial autocorrelation analysis to identify potential spatial clustering patterns of scarlet fever incidence rates within the research area, utilizing ArcGIS 10.8 [26]. Moran’s *I*, ranging from [−1] to [1], was used as the calculation index. Values above 0 denote positive spatial autocorrelation, where a value closer to 1 indicates a higher level of clustering [27, 28]. Moreover, we employed local indicators of spatial association (LISA) to pinpoint disease clusters based on population-adjusted incidence and interrogate the spatial dependencies between specific spatial units and adjacent area. The LISA map was applied to display the local spatial autocorrelation characteristics, and the infected region could further be divided into four types: High-High (HH), High-Low (HL), Low-High (LH), and Low-Low (LL). Specifically, HH and LL clusters reflected regions of spatial similarity, commonly known as hot-spots and cold-spots, whereas HL and LH outliers indicated areas that were distinctly different from their surroundings. HL outliers indicated isolated areas with high incidence surrounded by regions of low incidence, whereas low-high clusters marked low-incidence spots that are located near high-incidence zones. Statistical significance for spatial patterning was determined using a significance threshold of 0.05.

The spatiotemporal scan statistic method developed by Kulldorff can identify high clustering areas with statistically significant incidence [29], with SaTScan software version 10.3.2. We utilized a discrete Poisson probability model. This model structure incorporated the subdistrict-level population-at-risk as an offset term, comparing the observed case numbers with population-derived expected cases. We carried out retrospective space-time scanning analysis with subdistricts (towns) and months serving as the spatial and temporal units, respectively. In accordance with established protocols [26, 30], the maximum spatial and temporal cluster sizes were set to 50% of the population at risk and 50% of the total study period, respectively. Additionally, 999 Monte Carlo simulations were performed. After scanning the incidence rates of scarlet fever at different times and in different geographic areas, the log-likelihood ratio (LLR) and relative risk (RR) were calculated. We assessed statistical significance through the log-likelihood ratio (LLR), while the relative risk (RR) was used to measure the risk level compared to the outside area. The cluster with the highest LLR were classified as the primary clusters, while other statistically significant windows (*p* < 0.05) were designated as secondary clusters.

## Results

### The overall trend of incidence in different phases

**Fig. 1:**
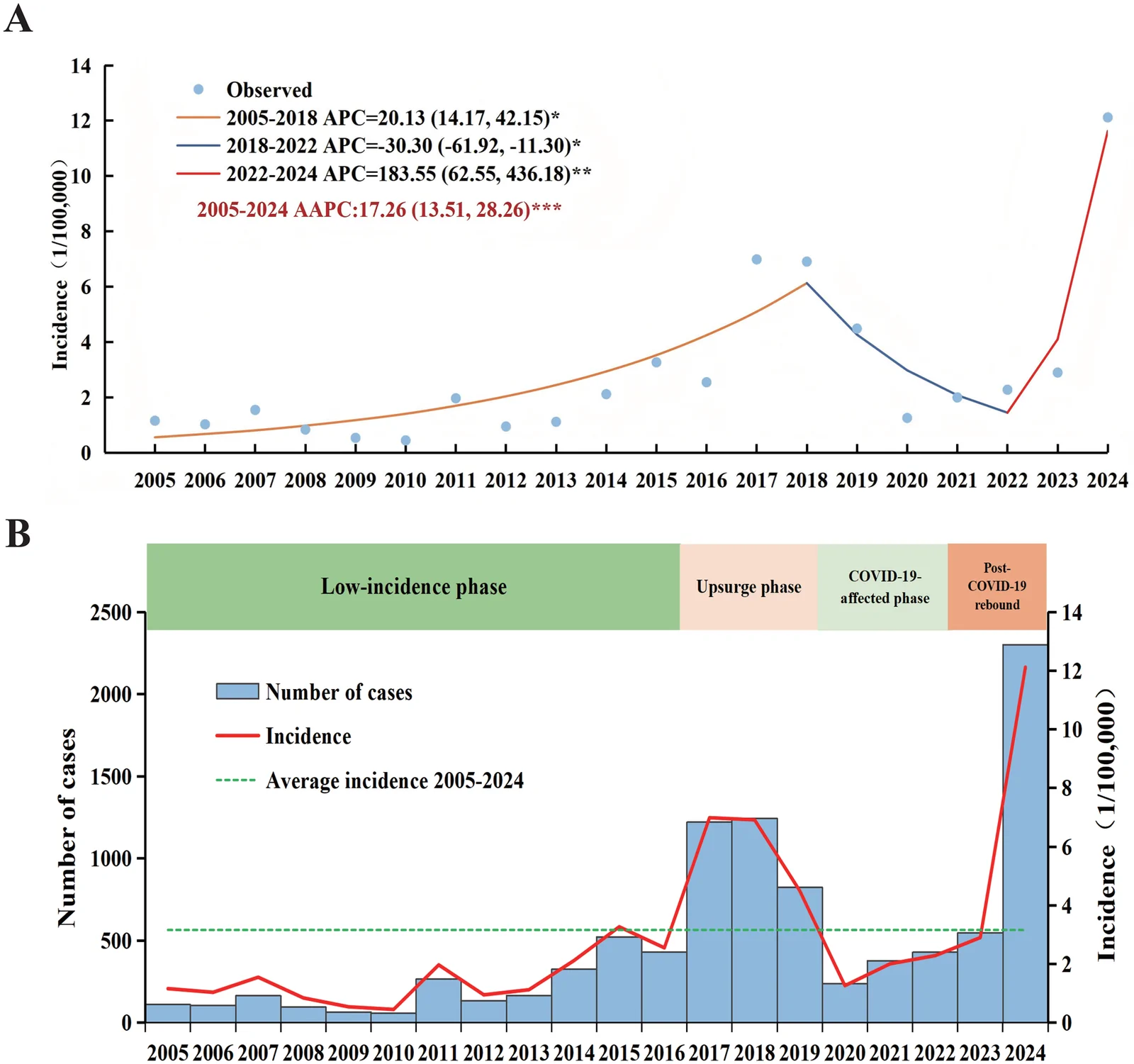
(**A**) the temporal trend of scarlet fever in Guangzhou from 2005 to 2024. The blue dots represent the observed raw annual incidence rates (per 100,000), and the solid lines indicate the predicted trend segments estimated by the Joinpoint model. APC: annual percent Change; AAPC: average annual percent change. (*p < 0.05, ** *p* < 0.01, ***p < 0.001). (**B**) annual incidence of scarlet fever in Guangzhou, 2005–2024

A total of 9,601 cases of scarlet fever were reported in Guangzhou from 2005 to 2024, including 517 laboratory-confirmed patients and 9,084 clinically diagnosed individuals (94.62%), with zero mortality. The average annual incidence rate of scarlet fever was approximately 3.16 per 100,000 people, of which the highest reported incidence rate in 2024 was 12.12 per 100,000 people, and the lowest incidence rate in 2010 was 0.45 per 100,000 people. Joinpoint regression analysis of the incidence level of scarlet fever in Guangzhou determined that there were two turning points in 2018 and 2022 (Fig. 1A). From 2005 to 2018, the incidence of scarlet fever showed an upward trend (APC = 20.13%; 95% CI: 14.17 - 42.15). This was followed by a decline from 2018 to 2022 (APC = −30.30%; 95% CI: −61.92 to −11.30). The incidence rate of scarlet fever rose again from 2022 to 2024 (APC = 183.55%, 95% CI: 62.55 - 436.18). The overall AAPC in incidence rate during the period from 2005 to 2024 was 17.26% (95% CI: 13.51 - 28.26).

To more accurately reflect the fluctuating dynamics of scarlet fever and to illustrate how COVID-19 has shaped scarlet fever trends, we have divided the research period into four distinct stages over time according to Joinpoint-identified inflections and key public health milestones (Fig. 1B). Specifically, the pre-COVID-19 phase (2005–2019) was partitioned into a low-incidence phase (2005–2016) and an upsurge phase (2017–2019), reflecting a distinct epidemiological shift where the average annual morbidity rose from a relative low level of 1.56 per 100,000 to a sustained high level of 6.11 per 100,000 people (range: 4.49 to 6.98 per 100,000 people). The nearly fourfold increase in the burden of the disease provided the basis of 2017–2019 as a distinct epidemiological period. During the pre-COVID-19 phase, cyclical epidemic fluctuations occurred 2 to 4 years, with peaks in 2007, 2011, 2015, and 2018 respectively. However, the trend was completely reversed in 2020, when the incidence rate of the disease fell sharply (1.26 per 100,000 people), followed by a minor escalation during 2021 – 2022. In summary, the incidence rate was relatively low with small fluctuations throughout the COVID-19-affected period (2020–2022), which can prove that it should be defined as an independent stage of epidemic control. Finally, the post COVID-19 rebound phase (2023–2024) refers to the stage after the relaxation of non-pharmaceutical interventions (NPIs). The annual incidence rate in 2023 also rose slightly, but it remained slightly below the average level. In 2024, the reported incidence rate surged sharply to 12.12 per 100,000 people, reaching an all-time high.

### Epidemiological characteristics

**Fig. 2:**
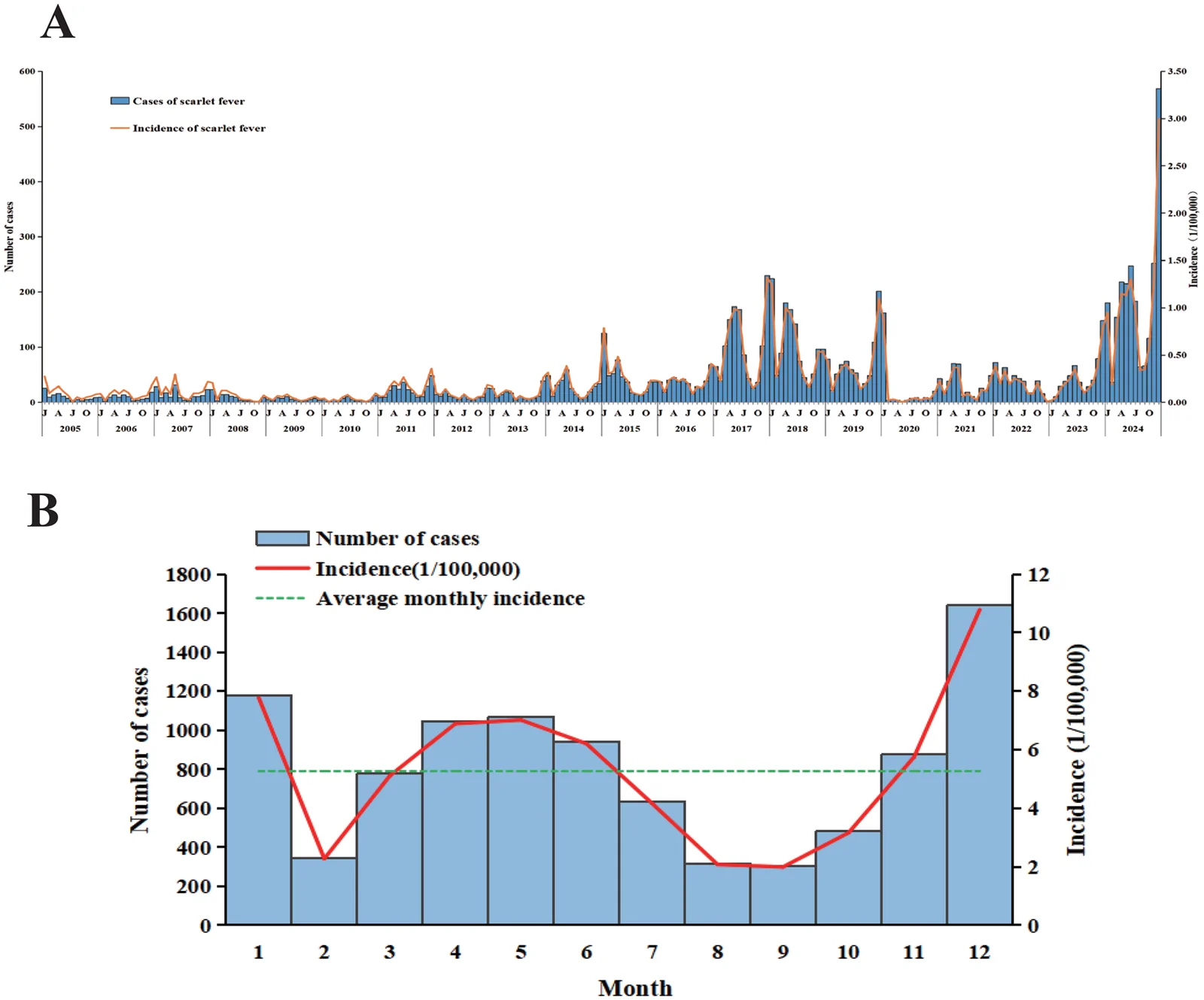
(**A**) monthly incidence and reported cases of scarlet fever. (**B**) monthly incidence of scarlet fever (12-month phase) in Guangzhou

From 2005 to 2024, scarlet fever cases persisted throughout the year, with infections documented in every month across Guangzhou (Fig. 2A). In addition to 2020, the incidence of scarlet fever has two seasonal peaks each year, from April to June (lower peak) and from November to January (higher peak) of the following year, of which the monthly incidence is highest in December (10.79 per 100,000 people). The seasonal nadir occurred in February and from August to September each year (Fig. 2B). However, in 2020, the epidemiological pattern of scarlet fever in Guangzhou deviated significantly from other years, characterized by the disappearance of the typical annual double-peak phenomenon.

Of these 9,601 cases, there were 6,169 male and 3,432 female, yielding a male-to-female ratio of 1.80:1. Scarlet fever cases were detected in all age groups, but the median age was 5 years old (ranging from 1 day to 70 years old). Individuals aged 10 years and below accounted for 97.46% of all cases, with the majority occurring among children 3–6 years old (66.12%), followed by those aged 7–10 years (24.56%) and 0–2 years (6.78%). Kindergarten children were the dominant infected population, accounting for 66.12% of the total number of patients, subsequently by students (26.47%), non-enrolled children (6.78%), and other populations (0.64%). Apart from gender, there were statistically significant differences in the proportion of cases among various age groups, occupational categories and administrative districts across different periods (all *p* < 0.001). Notably, the age group of 7 to 10 and the student group exhibited an upward trend, and the proportion of student cases reached 41.04% in 2023–2024, becoming a major affected group nearly comparable to kindergarten children (Table 1). Furthermore, the laboratory-confirmed cases accounted for 5.38% of the total, with an increasing trend observed across the four phases (*p* < 0.001).

**Table 1:** Characteristics of scarlet fever cases in Guangzhou, 2005–2024

	Total	2005–2016 ^a^	2017–2019 ^b^	2020–2022 ^c^	2023–2024 ^d^	P
N	9601	2428	3286	1041	2846	
Sex [n(%)]						0.756
Male	6169 (64.25)	1551 (63.88)	2100 (63.91)	666 (63.98)	1852 (65.07)	
Female	3432 (35.75)	877 (36.12)	1186 (36.09)	375 (36.02)	994 (34.93)	
Sex ratio	1.80	1.77	1.77	1.78	1.86	
Age groups [n(%)]						<0.001
0-	651 (6.78)	270 (11.12)	216 (6.57)	85 (8.17)	80 (2.81)	
3-	6348 (66.12)	1608 (66.23)	2427 (73.86)	725 (69.64)	1588 (55.80)	
7-	2358 (24.56)	443 (18.25)	572 (17.41)	217 (20.85)	1126 (39.56)	
11-	164 (1.71)	69 (2.84)	49 (1.49)	8 (0.77)	38 (1.34)	
15-	80 (0.83)	38 (1.57)	22 (0.67)	6 (0.58)	14 (0.49)	
Occupation [n(%)]						<0.001
Non-enrolled children	651 (6.78)	270 (11.12)	216 (6.57)	85 (8.17)	80 (2.81)	
Kindergarten children	6348 (66.12)	1608 (66.23)	2427 (73.86)	725 (69.64)	1588 (55.80)	
Students	2541 (26.47)	523 (21.54)	623 (18.96)	227 (21.81)	1168 (41.04)	
Others	61 (0.64)	27 (1.11)	20 (0.61)	4 (0.38)	10 (0.35)	
Category[n(%)]						<0.001
Clinical diagnosis	9084 (94.62)	2397 (98.72)	3230 (98.30)	1010 (97.02)	2447 (85.98)	
Laboratory-confirmed	517 (5.38)	31 (1.28)	56 (1.70)	31 (2.98)	399 (14.02)	
District [n(%)]						<0.001
Baiyun	2307 (24.03)	718 (29.57)	892 (27.15)	205 (19.69)	492 (17.29)	
Tianhe	1361 (14.18)	380 (15.65)	405 (12.33)	131 (12.58)	445 (15.64)	
Haizhu	1043 (10.86)	298 (12.27)	361 (10.99)	89 (8.55)	295 (10.37)	
Panyu	961 (10.01)	207 (8.53)	318 (9.68)	114 (10.95)	322 (11.31)	
Zengcheng	895 (9.32)	120 (4.94)	373 (11.35)	118 (11.34)	284 (9.98)	
Yuexiu	836 (8.71)	202 (8.32)	273 (8.31)	83 (7.97)	278 (9.77)	
Liwan	614 (6.40)	132 (5.44)	153 (4.66)	85 (8.17)	244 (8.57)	
Huangpu	566 (5.90)	138 (5.68)	164 (4.99)	68 (6.53)	196 (6.89)	
Huadu	560 (5.83)	170 (7.00)	192 (5.84)	60 (5.76)	138 (4.85)	
Nansha	238 (2.48)	17 (0.70)	83 (2.53)	41 (3.94)	97 (3.41)	
Conghua	220 (2.29)	46 (1.89)	72 (2.19)	47 (4.51)	55 (1.93)	

Data were expressed as mean (SD) for continuous variables and n (%) for categorical variables. ^a^ Low-incidence phase (2005–2016); ^b^ Upsurge phase (2017–2019); ^c^ COVID-19-affected phase (2020–2022); ^d^ Post-COVID-19 rebound phase (2023–2024)

### Geographical distribution in different phases

**Fig. 3:**
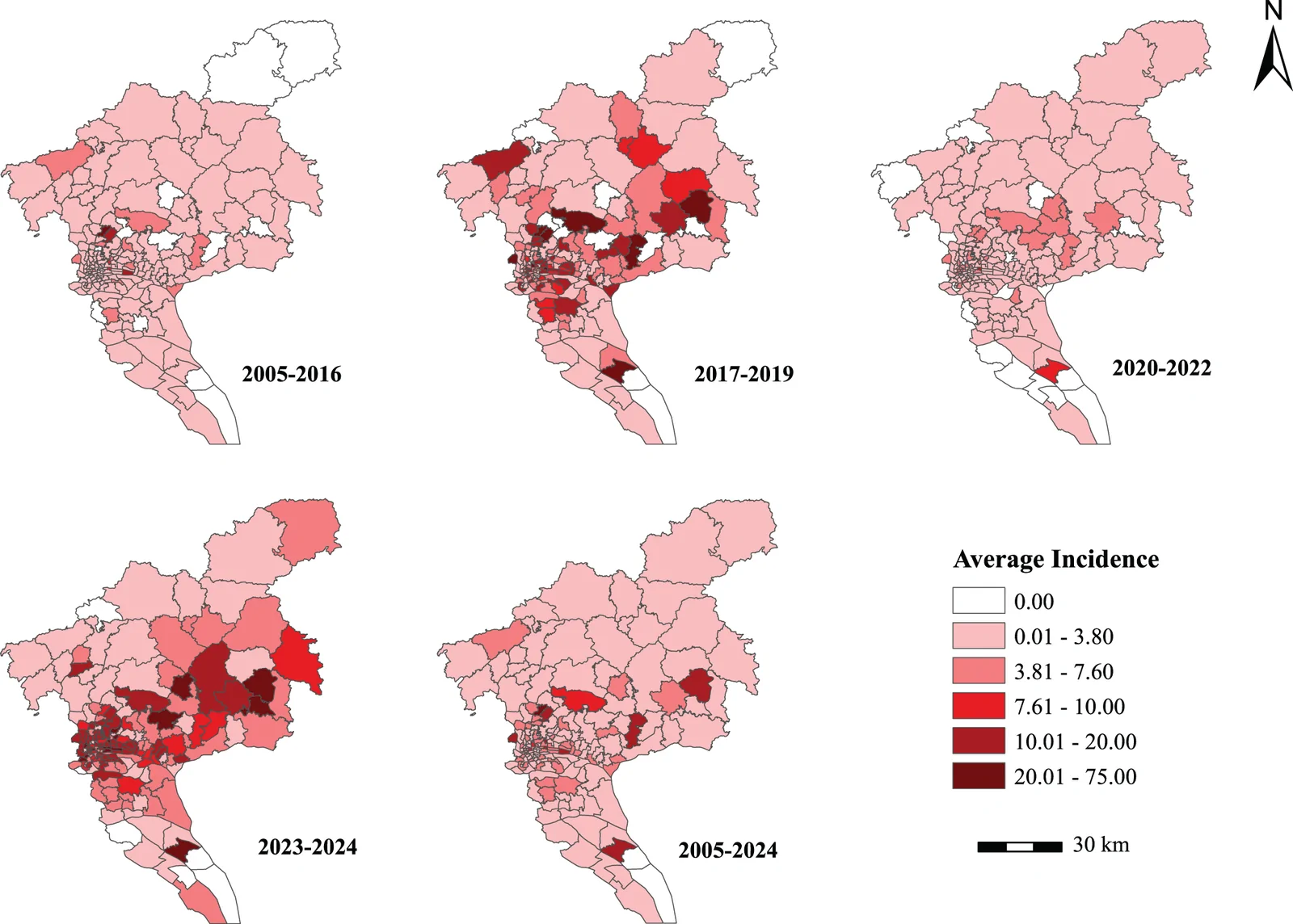
Spatiotemporal distribution of annual average incidence of scarlet fever at subdistrict (town) level in Guangzhou, 2005–2024

Eleven administrative districts in Guangzhou reported scarlet fever cases from 2005 to 2024, and high incidence regions were predominantly concentrated in the central region of Guangzhou (Supplementary Table 1 and Supplementary Fig. 2). The top five administrative regions with respect to the annual average reported incidence rate were Baiyun District (4.01 per 100,000 people), Yuexiu District (3.98 per 100,000 people), Tianhe District (3.84 per 100,000 people), Zengcheng District (3.64 per 100,000 people), and Haizhu District (3.20 per 100,000 people). These administrative districts accounted for 67.10% of the total number of reported cases. The proportional distribution of reported cases among regions differed significantly across different periods (*p* < 0.001) (Table 1). From 2005 to 2016, higher-morbidity areas were chiefly Guangzhou’s central urban districts and surrounding administrative districts. Between 2017 and 2024, while the incidence remained higher within the central urban districts and adjoining administrative districts, a significant expansion was observed toward expanding peri-urban areas, such as the Zengcheng District in the east. In addition, at the subdistrict (town) level, subdistricts characterized by elevated scarlet fever incidence rates were primarily located in Yuexiu (Guangta, Meihuacun and their surrounding areas), Baiyun (Jiahe, Yongping and their adjacent regions), and Tianhe (Yuancun, Shahe and their adjacent areas). Outside the urban core, Licheng and Lihu Subdistricts in Zengcheng District, along with Nansha Subdistrict in Nansha District, characterized as expanding peri-urban growth centers, have also emerged as focus areas (Fig. 3).

### Spatial autocorrelation analysis in different phases

The global autocorrelation outcomes of scarlet fever within Guangzhou were applied to detect the spatial clustering of the disease occurrence within four periods (Supplementary Table 2). Global Moran’s *I* values varied between 0.06 and 0.24, revealing that there is a certain degree of spatial autocorrelation between scarlet fever cases at the research stage. During the low-incidence, COVID-19-affected, and post-COVID-19 rebound phases, scarlet fever incidence exhibited statistically significant positive spatial autocorrelation (all *p* < 0.05) at the subdistrict (town) level.

**Fig. 4:**
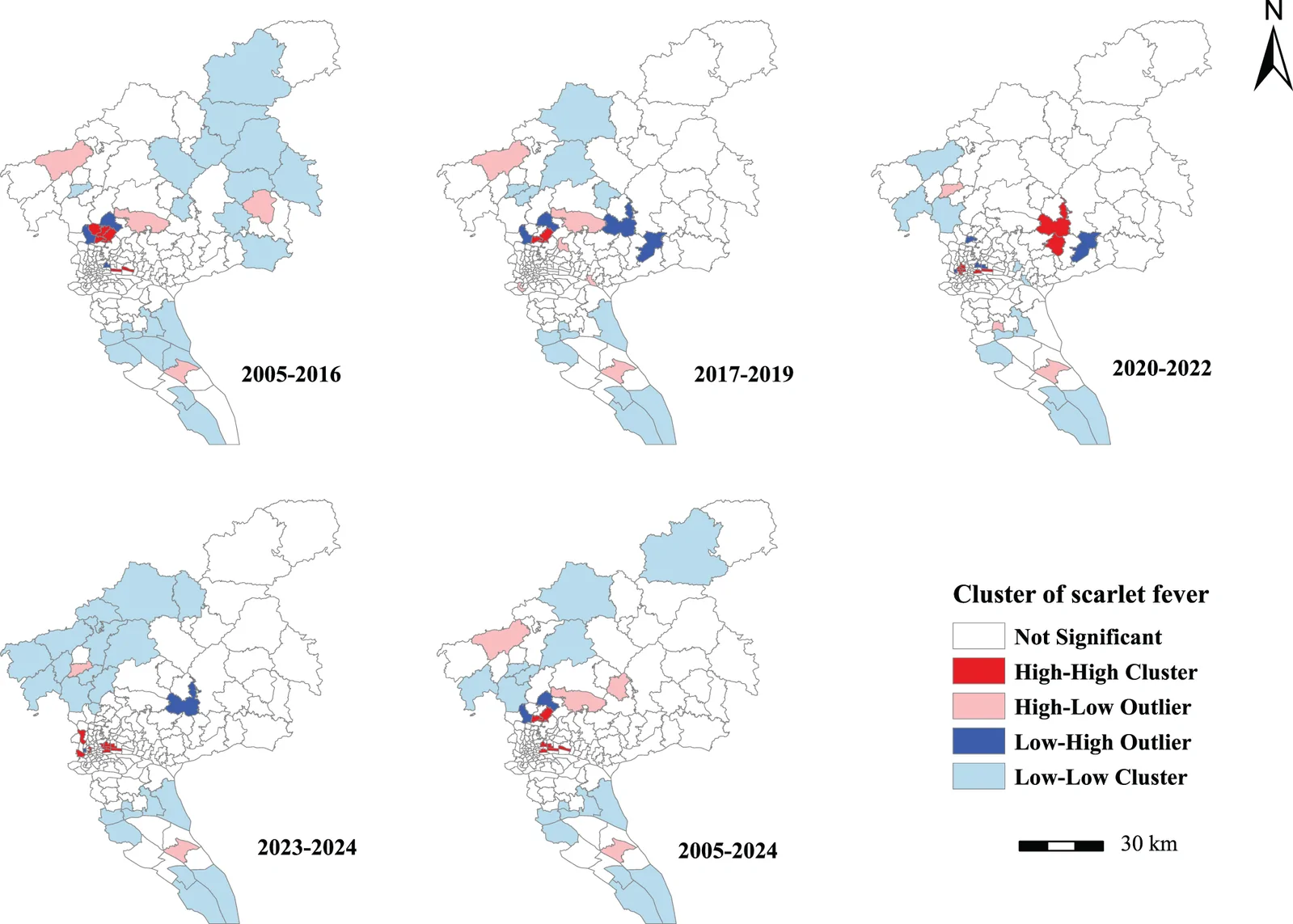
Results of the local spatial autocorrelation analysis of incidence of scarlet fever in Guangzhou, 2005–2024

Utilizing the average incidence data of scarlet fever in subdistricts (towns) of in Guangzhou from 2005 to 2024, the LISA indicated that hotspots (“High-High” clusters) appeared every year, and these hotspots were mainly concentrated in the central area of Guangzhou (Fig. 4). The high-high cluster involved Baiyun District (Jiahe, Yongping and Helong Subdistricts), Tianhe District (Tianyuan, Xiancun and Tianhenan Subdistricts), and Yuexiu District (Huanghuagang and Dongshan Subdistricts). Additionally, during the pre-COVID-19 phase, high-high clusters were mainly anchored in the aforementioned subdistricts of the Baiyun District, while high-density regions were predominantly aggregated in the streets of Yuexiu and Tianhe mentioned above in the COVID-19-affected and post-COVID-19 stages. To further examine annual variations, annual LISA maps (2005–2024) are provided in Supplementary Fig. 3. These annual maps demonstrated that while minor annual fluctuations occurred, the fundamental spatial hotspots remained highly consistent with our phase-specific findings. The high-low outliers included Taihe Town in Baiyun District, Longhu Subdistrict in Huangpu District, Nansha Subdistrict in Nansha District and Shiling Town in Huadu District. The low-high outliers included Baiyunhu and Longgui Subdistricts in Baiyun District. The low-low clusters were mainly concentrated in the northern and southern rural fringes of Guangzhou.

### Space-time scan statistical analysis

**Fig. 5:**
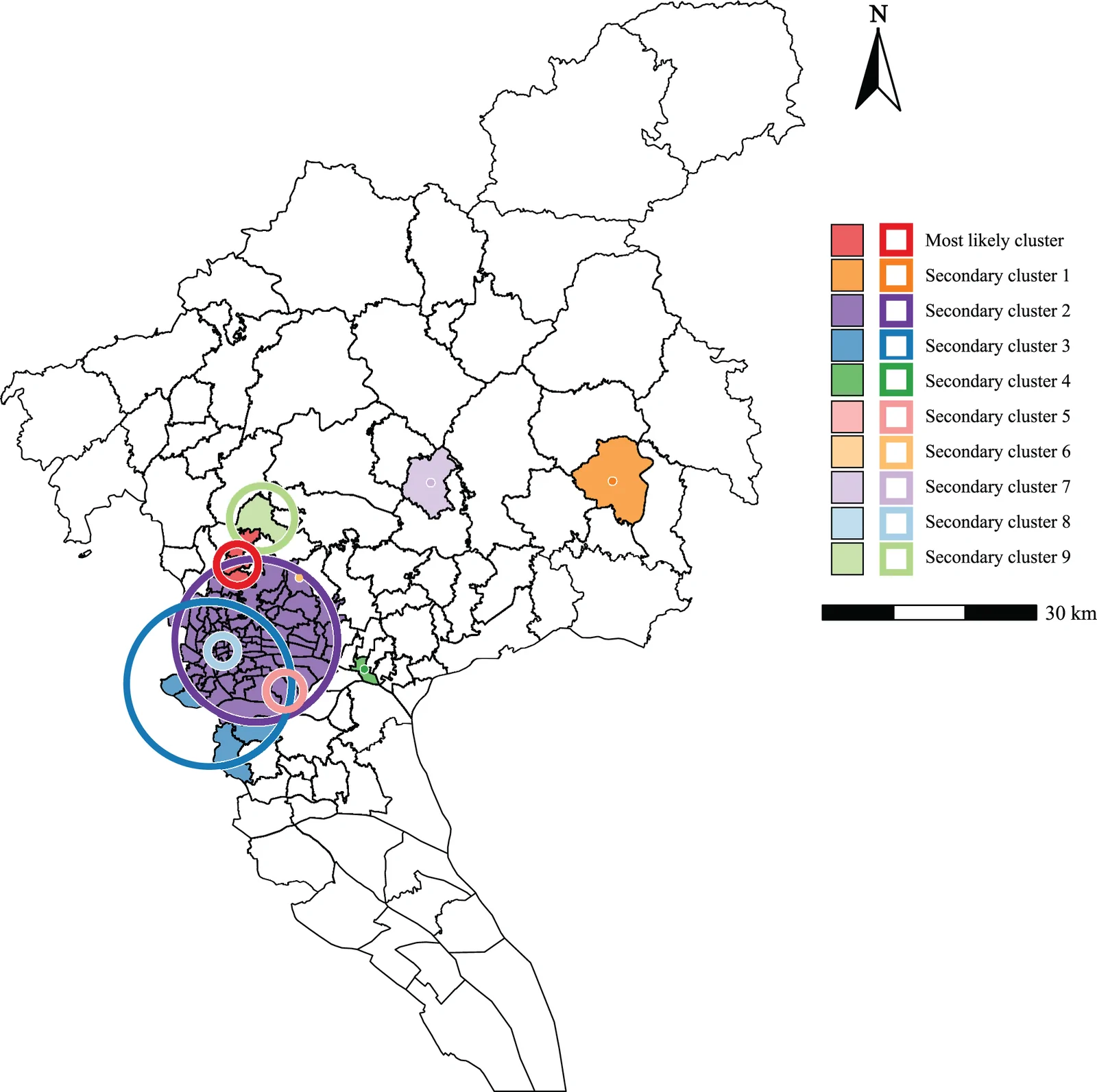
Spatiotemporal clusters of scarlet fever in Guangzhou, 2005–2024

The spatiotemporal scanning circle has accurately illustrated that the city center of Guangzhou has been the major gathering area in the past 20 years. We identified a primary high-risk cluster alongside nine secondary clustering zones within Guangzhou over the 2005–2024 period, using subdistricts (towns) as spatial units (all clusters with *p*-values < 0.05) (Table 2 and Fig. 5). Between December 2013 and January 2020, the primary spatiotemporal cluster covered 3 regions (Helong, Huangshi and Jiahe Subdistrict) of Baiyun District, with Helong Subdistrict (23.22°N, 113.28°E) at the central region, a circular zone with a 2.93 km radius. The Relative Risk (RR) was 32.43, indicating that the risk in this area was 32.43 times higher than in other areas. The most prominent secondary cluster occurred from November 2015 to December 2024, specifically involving the Licheng Subdistrict in Zengcheng District (23.33°N, 113.79°E), representing a key expanding peri-urban growth center in eastern Guangzhou, with an RR of 21.53. Additionally, from December 2016 to January 2020, and from December 2023 to December 2024, two large-scale secondary clusters predominantly covered the central urban zone and adjoining areas. One was centered at Meihuacun Subdistrict (23.13°N, 113.30°E) in Yuexiu District, comprising 88 subdistricts within the central urban areas, exhibiting a 6.79-fold increased risk. The other was centered at Baihedong Subdistrict (23.07°N, 113.24°E) in Liwan District, covering 69 subdistricts within the central urban areas, with an RR of 2.78.

**Table 2:** Retrospective spatial-temporal scan analysis of the incidence of scarlet fever in Guangzhou, 2005–2024

Cluster	Coordinates, radius (km)	Dates（Year-month）	Main areas in cluster (Subdistrict / Town)	RR	LLR	P
Most likely cluster	(23.22 N, 113.28 E), 2.93	2013.12–2020.1	Helong, Huangshi, Jiahe	32.43	1005.45	<0.001
Secondary cluster 1	(23.33 N, 113.79 E), 0	2015.11–2024.12	Licheng	21.53	475.65	<0.001
Secondary cluster 2	(23.13 N, 113.30 E), 11.42	2023.12–2024.12	Meihuacun, Dongshan, Nonglin, Huanghuagang, Baiyun, Tianhenan, Shahe, Dadong, Huale, Linhe, Xiancun (St), Datang, Liede, Jianshe, Dengfeng, Chigang, Shadong, Xingang, Binjiang, Zhuguang, Sushe, Shipai, Beijing, Hongqiao, Xinghua, Jiangnanzhong, Renmin, Fengyang, Changgang, Guangta, Haizhuang, Liuhua, Jingtai, Jianghai, Liurong, Wushan, Longjin, Jinhua, Longfeng, Hualin, Sanyuanli, Yuancun, Zhanqian, Lingnan, Yuangang, Tianyuan, Shayuan, Nanhuaxi, Fengyuan, Tangxia, Kuangquan, Ruibao, Shamian, Nanzhou, Caihong, Xicun, Nanshitou, Duobao, Tonghe, Nanyuan, Pazhou, Changxing, Changhua, Jingxi, Yuncheng, Huazhou, Tangjing, Huadi, Guanzhou, Tongde, Chongkou, Chebei, Baihedong, Qiaozhong, Dongsha, Huangshi, Shiweitang, Songzhou, Chajiao, Xinshi, Luopu, Dongjiao, Huangcun, Longdong, Qianjin, Helong, Xintang (St), Jinsha	6.79	423.78	<0.001
Secondary cluster 3	(23.07 N, 113.24 E), 11.58	2016.12–2020.1	Baihedong, Chongkou, Dongjiao, Dongsha, Shayuan, Nanshitou, Chajiao, Longfeng, Huadi, Zhongnan, Nanhua Xi, Shamei, Haichuang, Changgang, Jiangnanzhong, Lingnan, Duobao, Hailong, Ruibao, Hualin, Shiweitang, Renmin, Sushe, Changhua, Binjiang, Fengyang, Fengyuan, Longjin, Guangta, Xingang, Zhuguang, Jinhua, Beijing, Caihong, Liurong, Qiaozhong, Datang, Nanyuan, Dadong, Luopu, Baiyun, Nanzhou, Zhanqian, Hongqiao, Dongshan, Jianshe, Nonglin, Liuhua, Xicun, Chigang, Huale, Meihuacun, Dashi, Jianghai, Kuangquan, Huanghuagang, Dengfeng, Shibi, Liede, Songzhou, Tongde, Jinsha, Shahe, Huazhou, Tianhenan, Xiancun (St), Sanyuanli, Jingtai, Linhe	2.78	148.71	<0.001
Secondary cluster 4	(23.09 N, 113.45 E), 0	2017.5–2020.1	Huangpu	15.31	37.61	<0.001
Secondary cluster 5	(23.07 N, 113.34 E), 2.63	2014.5–2016.6	Huazhou, Guanzhou, Jianghai	12.25	31.68	<0.001
Secondary cluster 6	(23.21 N, 113.36 E), 0	2011.3–2012.3	Longdong	26.22	20.73	<0.001
Secondary cluster 7	(23.33 N, 113.54 E), 0	2021.7–2023.10	Longhu	16.97	18.89	0.002
Secondary cluster 8	(23.12 N, 113.26 E), 2.14	2006.1–2008.6	Renmin, Guangta, Haichuang, Zhuguang, Beijing, Longjin, Hualin, Lingnan, Nanhuaixi, Binjiang, Shamian, Jiangnanzhong	3.92	18.53	0.002
Secondary cluster 9	(23.28 N, 113.31 E), 4.55	2012.12–2013.6	Longgui, Jiahe	13.38	18.34	0.003

## Discussion

Utilizing a two-decade dataset derived from infectious disease surveillance, our study has revealed the epidemiological changes of scarlet fever in Guangzhou, from a decade of relative low-level stability (2005 to 2016) to a highly unstable period (2017 to 2024), eventually reaching a historic peak. This transformation reflected the readjusted ecological balance shaped by long-term evolutionary trends and the impact of the COVID-19 pandemic. By integrating these temporal phases with seasonal, demographic and spatial insights, our research provided a solid framework for responding to the ever-changing challenges of scarlet fever in the post-epidemic era.

From 2005 to 2018, scarlet fever in Guangzhou followed a distinct periodicity peaking at intervals of 2 to 4 years (notably in 2007, 2011, 2015, and 2018), consistent with national trends [31, 32]. At this stage, the average annual incidence of scarlet fever in Guangzhou is higher than that of Guangdong Province, but lower than high-burden regions like Beijing, Tianjin, Shanghai, Jiangsu, Zhejiang and most northwest, north, and northeast provinces in China. Due to strict measures of prevention and control [33], the incidence of the disease in Guangzhou was generally in a relatively quiescent period like most provinces during the COVID-19 phase. However, during the post COVID-19 rebound phase, as these measures were relaxed, the annual morbidity rate of scarlet fever in Guangzhou is higher than that in most provinces, except for Tianjin, Qinghai, Ningxia and Xinjiang. Moreover, we found that Guangzhou witnessed a sharp resurgence of scarlet fever in 2024 (12.12 per 100,000 people), exceeding historical levels. Many factors may contribute to the rebound. Primarily, during the COVID-19 non-pharmaceutical interventions (NPIs) period, the long-term relatively low incidence may lead to a decline in immunity of the population, thus forming an “immune debt”, which may increase the number of susceptible people [34]. After the lifting of NPIs, the resumption of crowd gathering activities, especially in key places such as nurseries and schools, is likely to increase the risk of transmission [19, 35]. Furthermore, the disease has entered a cyclical peak. In China, the reported incidence of scarlet fever peaks every 2 to 4 years on average [31]. Although the peaks of the last major cycle occurred in 2017 and 2018, the expected subsequent peak may be suppressed and delayed by extensive restrictions imposed during the COVID-19 pandemic. Once NPIs were completely relaxed, this increased susceptibility, coupled with the resumption of gatherings in schools and the natural 2–4 year cycle [31], may facilitate the intense and delayed rebound observed in 2024. Despite the 2–4 years cyclicality suggesting a potential decline after the peak in the coming years, given that the overarching upward trend observed since 2005 remains a significant concern, scarlet fever will remain a long-term public health challenge for Guangzhou. Additionally, the 2023 edition of the Scarlet Fever Diagnosis and Treatment Guidelines (released in December 2023) relaxed diagnostic criteria by adding GAS nucleic acid and antigen detection as laboratory confirmation evidence. The widespread use of antigen, nucleic acid and multi-pathogen respiratory detection methods might increase the laboratory confirmed cases and capture mild infections that were previously underreported. Nevertheless, as the majority of cases in different periods of this study were still clinically diagnosed, the observed peak likely reflects a genuine epidemiological rebound, although the inherent risk of misdiagnosis with other exanthematous diseases remains a factor.

The incidence of scarlet fever in Guangzhou shows two annual peaks, from April to June (lower peak) and from November to the following January (higher peak), while February and August to September are low-incidence periods, in line with the overall trend in China and several provinces within it [19, 21, 26, 36, 37]. This pattern is potentially driven by multiple natural and social factors such as climate conditions, population density, as well as the start and end times of nurseries and schools. Meteorological conditions, including favorable temperature and humidity in winter and spring may facilitate the survival of pathogens, while collective activities during the semester may further increase the risk of transmission [31]. However, in 2020, this pattern disappeared likely due to prevent and control the spread of the novel coronavirus. During this period, educational activities in Guangzhou transitioned to remote learning or were suspended entirely, and even if schools in Guangzhou reopened later, students must abide by stringent rules to prevent and control the spread of respiratory infectious diseases [38]. Additionally, the monthly incidence in December reached the highest value, and the monthly incidence of scarlet fever in December 2024 set a record in the city’s history. This suggests the importance of reinforcing winter precautions and maintaining continuous surveillance to monitor the evolving epidemiological landscape of scarlet fever. In addition, we observed that kindergarten children aged 3–6, especially men, emerged as the most susceptible population to scarlet fever within Guangzhou, which was consistent with evidence from other studies [6, 13, 21, 26, 37, 39]. This may be related to the poor personal hygiene habits and imperfect development of the autoimmune system in children aged 3 to 6, as well as the poor personal hygiene habits and frequent activities of male children [32]. It is worth noting that the proportion of cases among students, particularly those aged 7–10 years, increased substantially, becoming a major affected group nearly comparable to kindergarten children in 2023–2024, indicating an increased risk of cluster transmission among the student population, especially the group of primary school students in the lower grades. This may be because students, particularly those aged 7–10 years, had larger ‘immunity gap’, who missed critical years of natural exposure that would typically bolster population-level immunity, unlike younger cohorts who were just entering their susceptible window. Furthermore, after the full resumption of classes and the relaxation of NPIs, many susceptible school-age children gathered on campus and interacted with each other, providing more opportunities for cluster transmission compared to the more strictly supervised environment of childcare institutions [34, 35]. There is a need to reinforce the training and supervision of key places such as childcare institutions and primary schools before the peak of winter and spring, strictly implement morning and afternoon inspection and disinfection measures, strengthen health education, and optimize health resources to lean towards susceptible groups such as children aged 3–10 [20, 32].

Spatial distribution analysis indicated the scarlet fever epidemic in Guangzhou was characterized by a persistent concentration within central urban districts, with an expansion toward expanding peri-urban growth areas such as Zengcheng District in the east. The feature was in line with the common pattern of “higher incidence in the central urban areas” observed in major cities in China [19, 21, 26]. At the subdistrict (town) level, elevated incidence was not only sustained in established central hubs, such as Guangta in Yuexiu, Yuancun in Tianhe and Yongping in Baiyun, but also emerged prominently in expanding peri-urban growth centers, including Licheng in Zengcheng and Nansha in Nansha. The LISA further confirmed that ‘High-High’ clusters remained anchored within central urban subdistricts, including Xiancun in Tianhe, Huanghuagang and Dongshan in Yuexiu, as well as Yongping in Baiyun. Besides, space-time scanning revealed a dynamic shift in the clustering areas. The clusters were primarily concentrated in the Baiyun District of Helong, Huangshi, and Jiahe Subdistricts during the pre-COVID-19 period, while a sustained expansion toward expanding peri-urban growth centers like the Licheng Subdistrict in Zengcheng District, alongside a resurgence across the traditional central urban districts of Yuexiu, Liwan, Tianhe and Haizhu in the COVID-19-affected and post-COVID-19 phases. The combination of these spatial and temporal analyses indicated that these areas were persistent high-incidence hotspots with significant spatial clustering, representing key epidemic foci for GAS transmission. This might be the result of multiple factors working together, such as higher population density, high population mobility, concentrated educational resources, convenient transportation, and higher accessibility to medical services [19, 26]. These findings call for intensified public health interventions targeting scarlet fever in high-incidence subdistricts in the aforementioned central urban areas and expanding peri-urban growth centers of Guangzhou, to enhance monitoring and early warning, and to allocate more medical resources to these high-risk regions.

Our research had some limitations. As with any passive monitoring system, there may be many mild cases and asymptomatic infections in the community that have not been detected, which may result in the actual level being underestimated or omitted. Although a large part of the reported cases was clinically diagnosed in accordance with national surveillance standards, this symptomatic identification method might introduce the potential risk of misdiagnosis with other similar exanthematous diseases. Furthermore, while we retained the *p* < 0.05 threshold without FDR correction to prioritize the sensitivity of hot-spot detection, this approach may result in a slight overestimation of spatial clustering. The survey also lacked variables such as virus strain variation, host-level factors (e.g., household size and time to seek care), behavioral covariates, and environmental indicators (e.g., air quality and specific school-level factors), which might limit our ability to fully clarify the micro-level driving mechanisms of the epidemic. Future studies with comprehensive multi-source data are needed to explore the determinants of scarlet fever transmission. Nevertheless, this research provides a comprehensive long-term perspective covering 2005 to 2024 at a fine spatial-temporal scale. By mapping the evolution of high-risk areas and recording the changes in the disease burden from kindergarten children to school-age students particularly those aged 7–10 years after the COVID-19 pandemic, our research results provide a practical basis for the rational allocation of resources and the formulation of targeted interventions in high-density cities like Guangzhou.

## Conclusions

This study reveals a significant spatial-temporal clustering of scarlet fever in Guangzhou from 2005 to 2024, characterized by an overall upward trend and a notable recent resurgence. The high-risk regions were predominantly localized within the central urban regions and expanding peri-urban growth centers, such as Helong, Licheng, Meihuacun, and their surrounding regions. The outbreak mostly affects kindergarten children aged 3–6 years and students aged 7–10 years, with winter and spring as peaks. Control strategies should target high-risk seasons, populations and regions to reduce scarlet fever cases.

## Electronic supplementary material

Below is the link to the electronic supplementary material.

Supplementary material 1

## Data Availability

The raw data supporting this research remains under the custody of the Guangzhou CDC and is subject to strict usage restrictions. Consequently, they are not publicly releasable and made available to the authors solely for this study.

## Abbreviations

GAS A: Group A streptococcus
NNIDSS: National Notifiable Infectious Disease Surveillance System
APC: Annual percent change
AAPC: Average annual percent change
LISA: Local indicators of spatial association

## References

[1] Andrey DO, Posfay-Barbe KM. Re-emergence of scarlet fever: old players return? Expert Rev Anti Infect Ther. 2016;14(8):687–89. 10.1080/14787210.2016.1195684. 27249582

[2] Gajdács M, Ábrók M, Lázár A, Burián K. Beta-haemolytic group A, C and G streptococcal infections in southern Hungary: A 10-year population-based retrospective survey (2008–2017) and a review of the literature. IDR. 2020;13:4739–49. 10.2147/idr.S279157. PMC7781025 33408489

[3] Thacharodi A, Hassan S, Vithlani A, Ahmed T, Kavish S, Geli Blacknell NM, et al. The burden of group a Streptococcus (GAS) infections: the challenge continues in the twenty-first century. iScience. 2025;28(1):111677. 10.1016/j.isci.2024.111677. 39877071 PMC11773489

[4] Lu JY, Chen ZQ, Liu YH, Liu WH, Ma Y, Li TG, et al. Effect of meteorological factors on scarlet fever incidence in Guangzhou city, southern China, 2006–2017. Sci Total Environ. 2019;663:227–35. 10.1016/j.scitotenv.2019.01.318. 30711589

[5] Ohashi A, Murayama MA, Miyabe Y, Yudoh K, Miyabe C. Streptococcal infection and autoimmune diseases. Front Immunol. 2024;15:1361123. 10.3389/fimmu.2024.1361123. 38464518 PMC10920276

[6] Liu Y, Chan TC, Yap LW, Luo Y, Xu W, Qin S, et al. Resurgence of scarlet fever in China: a 13-year population-based surveillance study. The Lancet Infect Dis. 2018;18(8):903–12. 10.1016/s1473-3099(18)30231-7. 29858148 PMC7185785

[7] Bloom DE, Carapetis J. Strep A: challenges, opportunities, vaccine-based solutions, and economics. NPJ Vaccines. 2024;9(1):80. 10.1038/s41541-024-00863-7. 38641634 PMC11031564

[8] Kong D, Pan H, Wu H, Chen J. Engaging broader stakeholders to accelerate group a Streptococcus vaccine development. Vaccines (Basel). 2025;13(7):734. 10.3390/vaccines13070734. 40733711 PMC12299777

[9] Basetti S, Hodgson J, Rawson TM, Majeed A. Scarlet fever: a guide for general practitioners. Lond J Prim Care (Abingdon). 2017;9(5):77–79. 10.1080/17571472.2017.1365677. PMC5649319 29081840

[10] Quinn RW. Comprehensive review of morbidity and mortality trends for rheumatic fever, streptococcal disease, and scarlet fever: the decline of rheumatic fever. Clin Infect Dis. 1989;11(6):928–53. 10.1093/clinids/11.6.928. 2690288

[11] Wong SSY, Yuen KY. The comeback of scarlet fever. EBioMedicine. 2018;28:7–8. 10.1016/j.ebiom.2018.01.030. 29396303 PMC5835575

[12] Brockmann SO, Eichner L, Eichner M. Constantly high incidence of scarlet fever in Germany. The Lancet Infect Dis. 2018;18(5):499–500. 10.1016/s1473-3099(18)30210-x. 29695362

[13] Lamagni T, Guy R, Chand M, Henderson KL, Chalker V, Lewis J, et al. Resurgence of scarlet fever in England, 2014–16: a population-based surveillance study. The Lancet Infect Dis. 2018;18(2):180–87. 10.1016/s1473-3099(17)30693-x. 29191628

[14] Park DW, Kim SH, Park JW, Kim MJ, Cho SJ, Park HJ, et al. Incidence and characteristics of scarlet fever, South Korea, 2008–2015. Emerg Infect Dis. 2017;23(4):658–61. 10.3201/eid2304.160773. 28322696 PMC5367408

[15] Yung CF, Thoon KC. A 12 year outbreak of scarlet fever in Singapore. The Lancet Infect Dis. 2018;18(9):942. 10.1016/s1473-3099(18)30464-x. 30152353

[16] You Y, Qin Y, Walker MJ, Feng L, Zhang J. Increased incidence of scarlet fever — China, 1999−2018. China CDC Wkly. 2019;1(5):63–66. 10.46234/ccdcw2019.019. PMC8393181 34594606

[17] Carapetis JR, Steer AC, Mulholland EK, Weber M. The global burden of group a streptococcal diseases. The Lancet Infect Dis. 2005;5(11):685–94. 10.1016/s1473-3099(05)70267-x. 16253886

[18] Miller KM, Carapetis JR, Van Beneden CA, Cadarette D, Daw JN, Moore HC, et al. The global burden of sore throat and group a Streptococcus pharyngitis: a systematic review and meta-analysis. EClinicalMedicine. 2022;48:101458. 10.1016/j.eclinm.2022.101458. 35706486 PMC9124702

[19] Zhang X, You Y, Liu J, Sun L, Zhou H, Zhang X, et al. Effect of nonpharmaceutical interventions on the epidemic trends of scarlet fever in China: a population-based surveillance and modelling study. Int J Infect Dis. 2025;158:107969. 10.1016/j.ijid.2025.107969. 40582612

[20] Li K, Rui J, Song W, Luo L, Zhao Y, Qu H, et al. Temporal shifts in 24 notifiable infectious diseases in China before and during the COVID-19 pandemic. Nat Commun. 2024;15(1):3891. 10.1038/s41467-024-48201-8. 38719858 PMC11079007

[21] Wu R, Xiong Y, Wang J, Li B, Yang L, Zhao H, et al. Epidemiological changes of scarlet fever before, during and after the COVID-19 pandemic in Chongqing, China: a 19-year surveillance and prediction study. BMC Public Health. 2024;24(1):2674. 10.1186/s12889-024-20116-5. 39350134 PMC11443759

[22] National Health Commission of the People’s Republic of China. China Health Statistics Yearbook 2022. Beijing: National Health Commission of the People’s Republic of China. 2022.

[23] Liu YH, Wu D, Li XN, Li MX, Wang DH, Lu JY. Epidemiological characteristics of scarlet fever in Guangzhou from 2005 to 2020. J Med Pest Control. 2021;37(6):532-535. 10.7629/yxdwfz202106005.

[24] Yin SH, Ma MM, Ran R, Liu YH, Luo L. Epidemiological characteristics of scarlet fever in Guangzhou from 2010 to 2023. South China J Preventative Med. 2025;51(3):319–322. 10.12183/j.scjpm.2025.0319.

[25] Worldpop. Open spatial demographic data and research. Worldpop. 2023. https://www.worldpop.org/. 2025 Dec 15.

[26] Yu W, Guo L, Shen X, Wang Z, Cai J, Liu H, et al. Epidemiological characteristics and spatiotemporal clustering of scarlet fever in Liaoning Province, China, 2010–2019. Acta Trop. 2023;245:106968. 10.1016/j.actatropica.2023.106968. 37307889

[27] Kianfar N, Taghi M, Dasdar S, Mollalo A, Kiani B. Geospatial and machine learning analyses of cardiovascular disease mortality across the continental United States: identifying associated variables using Shapley values. BMC Public Health. 2026;26(1). 10.1186/s12889-026-26571-6. PMC12983589 41656207

[28] Kianfar N, Mesgari MS. GIS-based spatio-temporal analysis and modeling of COVID-19 incidence rates in Europe. Spatial Spatio-temporal Epidemiol. 2022;41:100498. 10.1016/j.sste.2022.100498. PMC8894707 35691655

[29] Kulldorff M, Nagarwalla N. Spatial disease clusters: detection and inference. Stat Med. 1995;14(8):799–810. 10.1002/sim.4780140809. 7644860

[30] Mahara G, Wang C, Huo D, Xu Q, Huang F, Tao L, et al. Spatiotemporal pattern analysis of scarlet fever incidence in Beijing, China, 2005–2014. Int J Environ Res Public Health. 2016;13(1):131. 10.3390/ijerph13010131. 26784213 PMC4730522

[31] Cui JZ, Yewu S, Xuemei. Spatiotemporal scan statistic of scarlet fever in China, 2010−2019. Dis Surveillance. 2023;38(3):287–93. 10.3784/jbjc.202209300433.

[32] Liu S, Liu H, Huang Y, Lei X, Ke D, Lei Z, et al. In-depth spatio-temporal analysis of scarlet fever in subtropical China: Aiding targeted control efforts and the healthy China initiative. Acta Trop. 2026;273:107954. 10.1016/j.actatropica.2025.107954. 41448481

[33] Wu N, Guan P, An S, Wang Z, Huang D, Ren Y, et al. Assessing the impact of COVID-19 non-pharmaceutical interventions and relaxation policies on class B respiratory infectious diseases transmission in China. Sci Rep. 2024;14(1):21197. 10.1038/s41598-024-72165-w. 39261569 PMC11390917

[34] Cohen R, Ashman M, Taha MK, Varon E, Angoulvant F, Levy C, et al. Pediatric infectious disease group (GPIP) position paper on the immune debt of the COVID-19 pandemic in childhood, how can we fill the immunity gap? Infect Dis Now. 2021;51(5):418–23. 10.1016/j.idnow.2021.05.004. 33991720 PMC8114587

[35] Sultana Q, Agrawal V, Jaiswal V, Mohanty A, Sah R. Was the COVID pandemic suppressing the outbreak of scarlet fever in the United Kingdom?: correspondence. Int J Surg. 2023;109(3):626–28. 10.1097/js9.0000000000000225. 36906776 PMC10389450

[36] Yang T, Du X, Li J, Zhang T, Wang Y, Wang L. Modeling transmission dynamics and socio-economic determinants of scarlet fever in Chengdu, China: an integrated SEIAR and machine learning approach. Epidemics. 2025;52:100844. 10.1016/j.epidem.2025.100844. 40651089

[37] Zhang Q, Liu W, Ma W, Shi Y, Wu Y, Li Y, et al. Spatiotemporal epidemiology of scarlet fever in Jiangsu Province, China, 2005–2015. BMC Infect Dis. 2017;17(1):596. 10.1186/s12879-017-2681-5. 28854889 PMC5576110

[38] Lai S, Ruktanonchai NW, Zhou L, Prosper O, Luo W, Floyd JR, et al. Effect of non-pharmaceutical interventions to contain COVID-19 in China. Nature. 2020;585(7825):410–13. 10.1038/s41586-020-2293-x. 32365354 PMC7116778

[39] Chen H, Chen Y, Sun B, Wen L, An X. Epidemiological study of scarlet fever in Shenyang, China. BMC Infect Dis. 2019;19(1):1074. 10.1186/s12879-019-4705-9. 31864293 PMC6925867

