# Supplementary material for: Epidemiological characteristics and spatiotemporal analysis of scarlet fever in Guangzhou, China: the surveillance of 20 years
Source: BMC Infect Dis. 2026 Jun 26;26:1646. doi: 10.1186/s12879-026-13770-0 (PMC13563735; doi:10.1186/s12879-026-13770-0)
Supplement: Supplementary file 1 [file 12879_2026_13770_MOESM1_ESM.docx]

**Additional Information** **Web Appendix**

**Supplementary Table 1.** Annual incidence of scarlet fever by administrative districts of Guangzhou (2005-2024).

| **Districts** | **Annualized mean incidence (per 100,000 people)** | | | | | **AAPC (95 % CI)^a^** |
| --- | --- | --- | --- | --- | --- | --- |
|  | **2005-2024** | **2005-2016** | **2017-2019** | **2020-2022** | **2023-2024** |  |
| Liwan | 3.03 | 1.20 | 4.32 | 2.43 | 10.76 | 13.99 (6.07, 31.27)*** |
| Yuexiu | 3.98 | 1.57 | 8.84 | 2.67 | 14.33 | 11.59 (3.71, 27.73)*** |
| Haizhu | 3.20 | 1.63 | 6.69 | 1.64 | 8.34 | 7.84 (-0.60, 24.68) |
| Tianhe | 3.84 | 2.14 | 6.30 | 1.95 | 9.92 | 8.35 (0.01, 24.08)* |
| Baiyun | 4.01 | 2.53 | 8.33 | 1.85 | 6.68 | 3.34 (-11.27, 31.91) |
| Huangpu | 2.89 | 1.41 | 4.45 | 1.86 | 7.98 | 9.69 (1.70, 25.00)** |
| Panyu | 2.25 | 0.97 | 4.24 | 1.37 | 5.68 | 8.64 (0.27, 27.00) |
| Huadu | 2.22 | 1.41 | 4.14 | 1.18 | 3.97 | 4.20 (-6.15, 24.75) |
| Nansha | 1.96 | 0.33 | 3.42 | 1.53 | 4.97 | 10.94 (-6.61, 52.49) |
| Conghua | 1.73 | 0.66 | 3.44 | 2.15 | 3.74 | 7.59 (-1.06, 23.27) |
| Zengcheng | 3.64 | 0.95 | 8.74 | 2.59 | 8.91 | 7.22 (-11.42, 49.47) |

^a^ AAPC: Average annual percentage change. (**P* < 0.05, ** *P* < 0.01, ****P* < 0.001).

**Supplementary Table 2.** Results of global spatial autocorrlation analysis for incidence of scarlet fever in Guangzhou, 2005–2024.

| **Year** | **Moran′s *I*** | **z-score** | ***P-value*** | ***q* value** | **Cluster** |
| --- | --- | --- | --- | --- | --- |
| 2005-2016 | 0.11 | 2.85 | 0.004 | 0.007 | Yes |
| 2017-2019 | 0.06 | 1.39 | 0.163 | 0.165 | No |
| 2020-2022 | 0.17 | 3.88 | <0.001 | <0.001 | Yes |
| 2023-2024 | 0.24 | 5.36 | <0.001 | <0.001 | Yes |
| 2005-2024 | 0.09 | 2.19 | 0.028 | 0.036 | Yes |

The *q* values (false discovery rate adjusted *P*-value) were calculated using the Benjamini-Hochberg method.

**Supplementary Fig. 1.** Administrative divisions and hierarchical structure of Guangzhou, China. (A) Spatial boundaries of the 11 districts; (B) Spatial boundaries of the subdistricts and towns.

**Supplementary Fig. 2.** Spatiotemporal distribution of annual average incidence of scarlet fever at district level in Guangzhou, 2005–2024.

**Supplementary Fig. 3.** Annual LISA cluster maps of scarlet fever in Guangzhou, 2005–2024.
